# Early detection of Angelman syndrome resulting from *de novo* paternal isodisomic 15q UPD and review of comparable cases

**DOI:** 10.1186/1755-8166-6-35

**Published:** 2013-09-08

**Authors:** Emese Horváth, Zsuzsanna Horváth, Dóra Isaszegi, Gyurgyinka Gergev, Nikoletta Nagy, János Szabó, László Sztriha, Márta Széll, Emőke Endreffy

**Affiliations:** 1 Department of Medical Genetics, University of Szeged, 4 Somogyi B. utca, H-6720, Szeged, Hungary; 2 Department of Pediatrics and Child Health Centre, University of Szeged, Szeged, Hungary; 3 Dermatological Research Group of the Hungarian Academy of Sciences, University of Szeged, Szeged, Hungary

**Keywords:** Angelman syndrome, Isodisomic 15, Uniparental disomy, Balanced translocation chromosome 15q

## Abstract

**Background:**

Angelman syndrome is a rare neurogenetic disorder that results in intellectual and developmental disturbances, seizures, jerky movements and frequent smiling. Angelman syndrome is caused by two genetic disturbances: either genes on the maternally inherited chromosome 15 are deleted or inactivated or two paternal copies of the corresponding genes are inherited (paternal uniparental disomy). A 16-month-old child was referred with minor facial anomalies, neurodevelopmental delay and speech impairment. The clinical symptoms suggested angelman syndrome. The aim of our study was to elucidate the genetic background of this case.

**Results:**

This study reports the earliest diagnosed angelman syndrome in a 16-month-old Hungarian child. Cytogenetic results suggested a *de novo* Robertsonian-like translocation involving both q arms of chromosome 15: 45,XY,der(15;15)(q10;q10). Molecular genetic studies with polymorphic short tandem repeat markers of the fibrillin-1 gene, located in the 15q21.1, revealed that both arms of the translocated chromosome were derived from a single paternal chromosome 15 (isodisomy) and led to the diagnosis of angelman syndrome caused by paternal uniparental disomy.

**Conclusions:**

AS resulting from paternal uniparental disomy caused by *de novo* balanced translocation t(15q;15q) of a single paternal chromosome has been reported by other groups. This paper reviews 19 previously published comparable cases of the literature. Our paper contributes to the deeper understanding of the phenotype-genotype correlation in angelman syndrome for non-deletion subclasses and suggests that patients with uniparental disomy have milder symptoms and higher BMI than the ones with other underlying genetic abnormalities.

## Background

Angelman syndrome (AS; OMIM 105830) is a rare neurodevelopmental disorder characterized by severe mental and physical delay, limited speech, fine tremor, ataxia, excessive mouthing behavior, fascination with water, jerky limb movements, seizures, craniofacial abnormalities and unusually happy sociable behavior characterized by frequent episodes of inappropriate smiling [1,2].

Seventy percent of AS cases investigated with molecular genetics methods are the result of a small deletion in the 11–13 region of the maternal chromosome 15. A deletion in the same region of the paternal chromosome 15 results in the sister disorder Prader-Willi syndrome (PWS). Expression of the genes in the 11–13 region is regulated by the PWS/AS imprinting center (IC), which differentially silences the paternal copy of the ubiquitin protein ligaseE3A (*UBE3A*) gene in the hippocampus and in the cerebellum. Other genetic abnormalities resulting in AS reported include uniparental disomy (UPD; 5%), mutations of the IC (5%), mutations of the UBE3A gene (10%), and other mechanisms (10%) [3,4].

**Figure 1:**
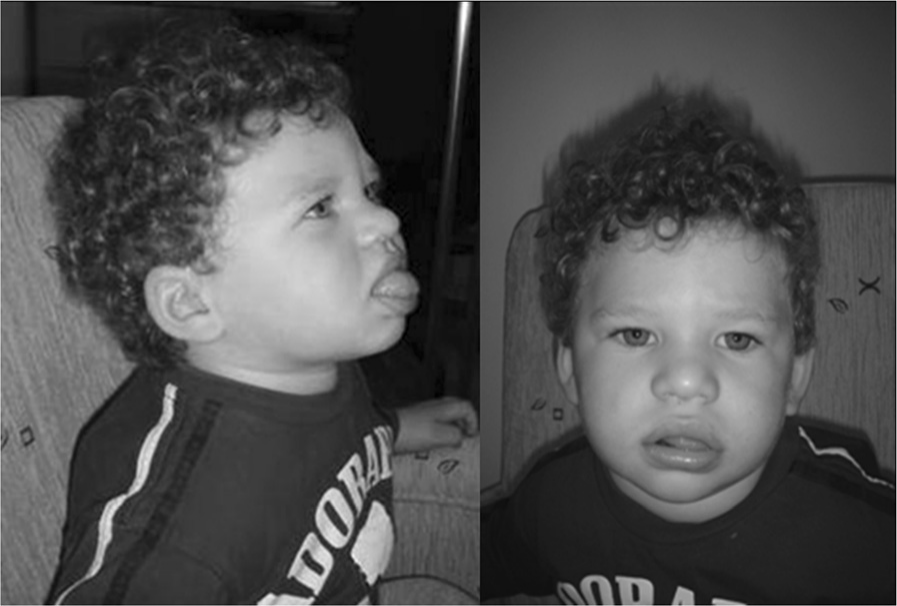
**Clinical features of a patient with Angelman syndrome resulting from *de novo *paternal isochromosome 15q UPD.** The dysmorphic symptoms of the 16 month old child include wide nasal bridge, low set ears, thick lips, wide mouth and protruding tongue.

In this paper, we report a 16-month-old Hungarian child, who was referred to our genetic counseling unit with delayed psychomotor and speech development and dysmorphic features, including wide nasal bridge, low set ears, thick lips, wide mouth with protuberant tongue (Figure 1). Tongue thrusts were observed. Head circumference was 47 cm (25 percentile). The affected child was born at term after an uneventful first pregnancy with normal weight (3260 g) and head circumference (33 cm). The Apgar scores were 9, 10 and 10 at 1, 5 and 10 minutes, respectively. No signs of decreased fetal movement, neonatal hypotonia or feeding difficulties were reported. The clinical phenotype of the patient suggested AS, therefore molecular cytogenetic investigations were carried out to elucidate the genetic background of the presented case.

## Results

**Figure 2:**
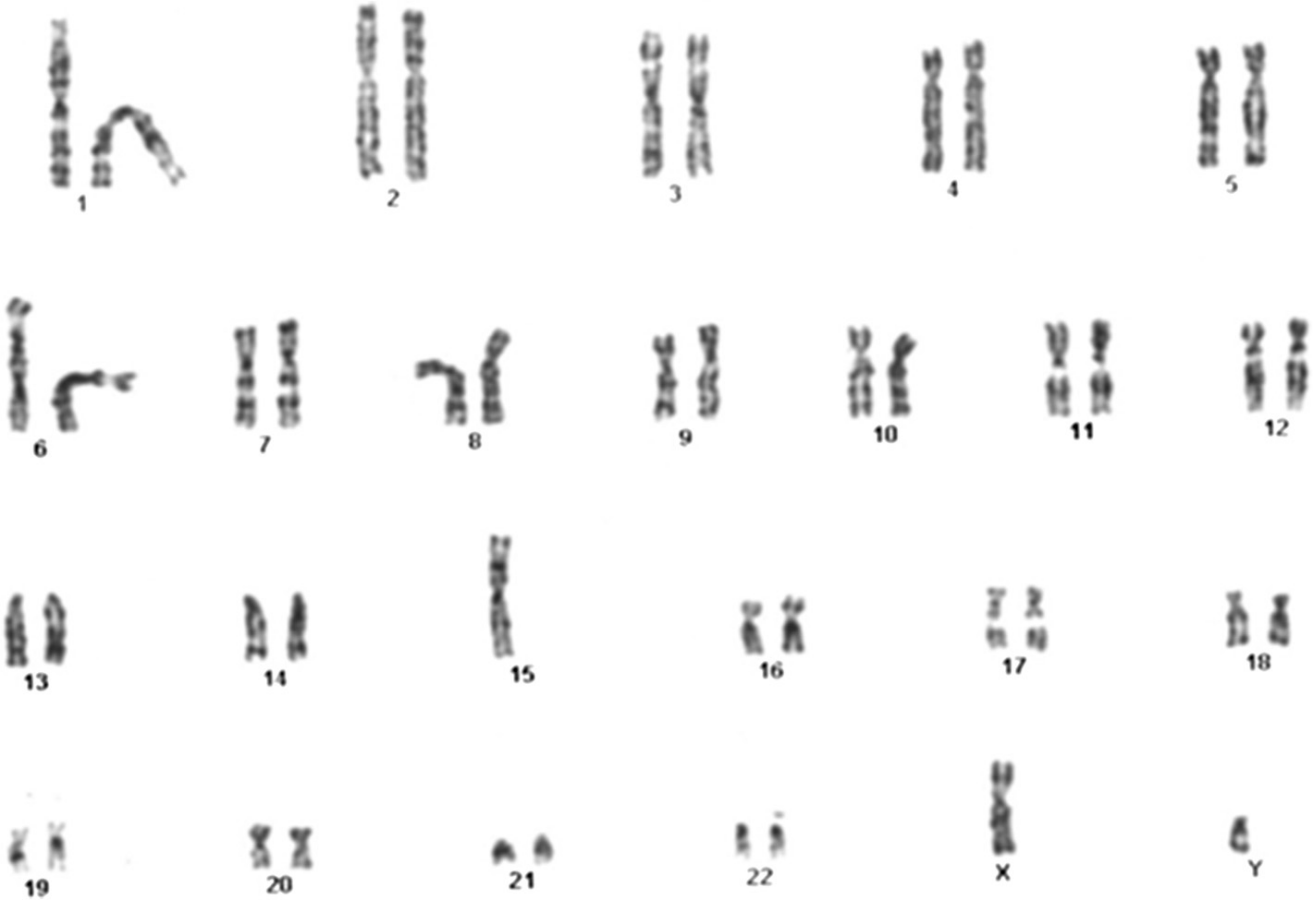
**The karyotype of the AS patient.** The cytogenetic image displays 45 metaphase chromosomes with an apparently balanced homologous rearrangement between the long arms of chromosomes 15. Cytogenetic result: 45,XY der(15;15)(q10;q10).

Cytogenetic analysis demonstrated a 45,XY,der(15;15)(q10;q10) karyotype in all analyzed cells from the index patient (III/1, Figure 2). All metaphase cells displayed 45 chromosomes, suggesting a balanced homologous rearrangement of the long arms of chromosomes 15. The parent’s karyotype was found to be normal, indicating a *de novo* chromosome rearrangement in the patient.

**Figure 3:**
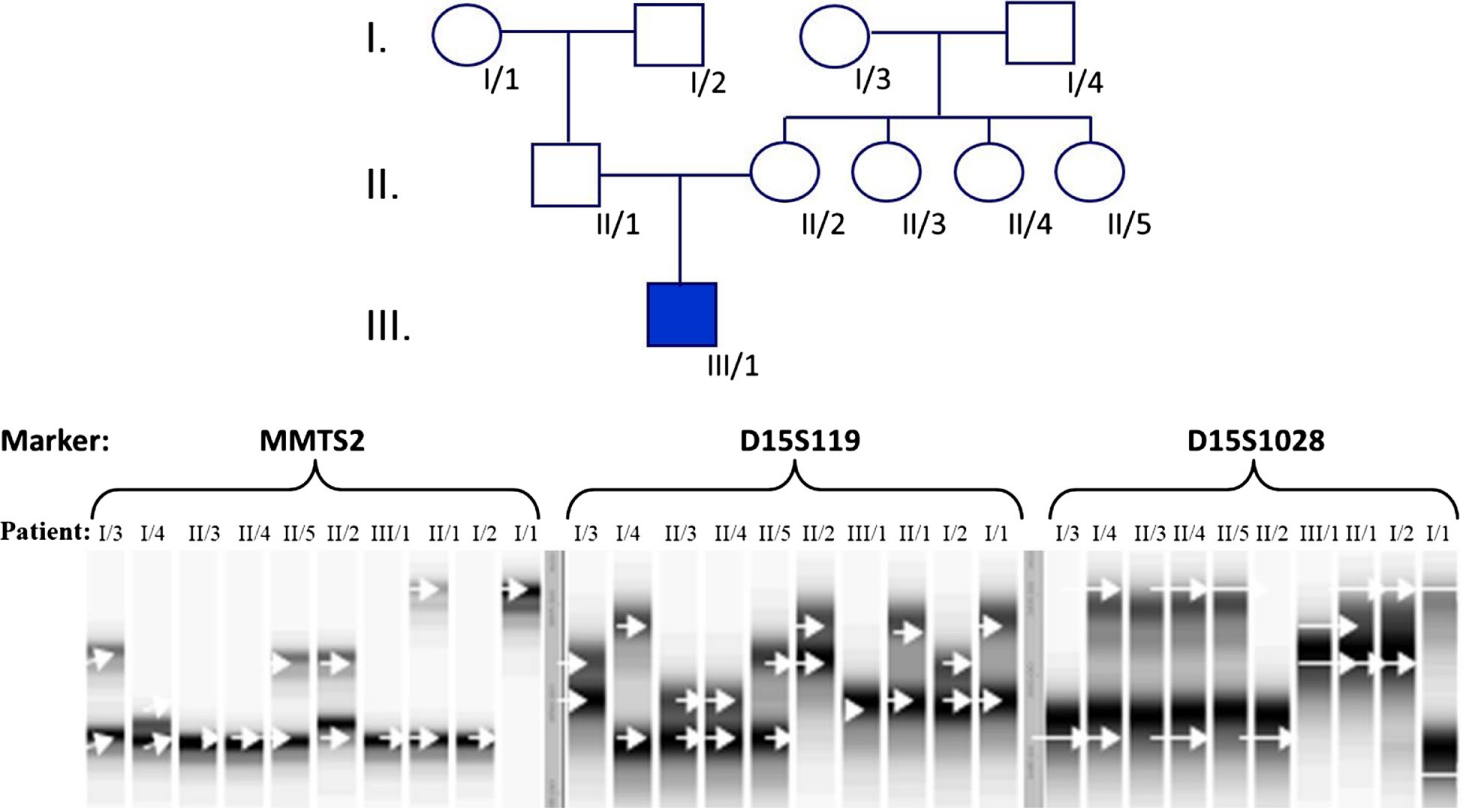
**Genetic analysis of the affected family using polymorphic STR markers MMTS2, D15S119 and D15S1028 for the fibrillin-1 gene.** Marker analysis of the patient (III/1), his parents (II/1, II/2), maternal aunts (II/3, II/4, II/5), maternal grandparents (I/3, I/4), and paternal grandparents (I/1, I/2) was performed by ALFexpress gel electrophoresis. The patient is homozygous for polymorphisms occurring in the father but not the mother, indicating that both arms of the aberrant chromosome 15 were of paternal origin.

Analysis of polymorphic short tandem repeat (STR) markers of the fibrillin-1 gene, which is located in 15q21.1, revealed that both long arms of the aberrant chromosome 15 were inherited from the father (Figure 3), allowing a diagnosis of AS caused by paternal UPD. The patient was homozygous at all loci for which his father was heterozygous, indicating that the rearrangement resulted from an isodisomic 15q.

## Discussion

Cytological and molecular genetic investigation revealed UPD suggesting a Robertsonian-like translocation 45,XY,der(15;15)(q10;q10), a rearrangement of the acrocentric chromosomes. Robertsonian translocations mostly form *de novo* due to intrinsic properties of the acrocentric chromosomes, which are likely to be the results of the high homology between the short arm DNA sequences of them [5]. A similar balanced 15;15 translocation resulting from paternal UPD in AS were reported by Freeman *et al.* (1993) [6], by Tonk *et al.* (1996) [7], by Ramsden *et al.* (1996) [8], by Guitart *et al.* (1997) [9], by Fridman *et al.* (1998) [10] and by Robinson *et al.* (2000) [11].

Results from polymorphic STR marker analysis for the fibrillin-1 gene, located in 15q21.1, indicated that both arms of the aberrant chromosome 15 were inherited from the father, allowing a diagnosis of AS caused by paternal UPD. DNA polymorphic markers demonstrated that the patient was homozygous at all loci for which the father was heterozygous, suggesting that the structural rearrangement was an isodisomic 15q and not a Robertsonian translocation. Similar cases of AS resulting from isodisomic 15q associated UPD have already been demonstrated by Freeman *et al.* (1993) [6] and by Robinson *et al.* (2000) [11], however, the majority of the previously reported paternal UPD associated AS cases were heterodisomic [7-10].

The severity of AS symptoms varies significantly. Bottani *et al.* (1994) were the first, who reported that the phenotype of AS with paternal isochromosome 15 is milder than those caused by other mechanisms [12]. This observation was confirmed by Tonk *et al.* (1996) [7], Smith *et al.* (1997) [13], Fridman *et al.* (1998) [10] and Moncla *et al.* (1999) [14], however Prassad *et al.* (1997) [15] have not observed differences between deletion and UPD, moreover Poyatos *et al.* (2002) described an even more severe phenotype [3]. The mildest symptoms have been reported for mutations of the UBE3A gene [2,12,14,16], whereas the most severe symptoms are reported for large deletions on chromosome 15 [2,14,16]. Varela *et al.* (2004) suggested that AS patients with UPD may remain undiagnosed because of their milder or less typical phenotype, leading to an overall under-diagnosis of the disease (Table 1) [17,18]. According to Tan *et al.* (2011) [4], 46% of AS children with UPD/imprinting defect showed significantly higher body mass index (BMI) than the ones carrying deletions.

**Table 1:** **The clinical features of the patient, in order of frequency, compared to the 13 AS patients with UPD/imprinting defects reported by Tan**[4]

**The analyzed parameters at diagnosis**	**Values for the patient described in this report**	**Values for the 13 AS patients with UPD/imprinting defects reported by Tan**[4]
Age (months) at diagnosis		
0–24	1	0
25–36	-	5
37–60	-	8
Gender	M	8M/5F
Short attention span	+	12/13 (92%)
History of sleep difficulties	+	12/13 (92%)
Normal tone at evaluation	+	12/13 (92%)
Mouthing behavior	+	11/13 (85%)
Hand flapping	+	11/13 (85%)
Drooling	+	10/13 (77%)
Feeding difficulties in infancy	-	10/13 (77%)
Ataxic or broad based gait	-	8/11 (73%)
Gastro-esophageal reflux	-	9/13 (69%)
Widely spaced teeth	+	9/13 (69%)
Fascination with water	+	8/13 (62%)
Easily provoked laughter	+	8/13 (62%)
Clinical seizures	-	6/13 (46%)
BMI>85%	+	6/13 (46%)
Unusually light hair or skin color	-	3/13 (23%)
Prognathism	-	3/13 (23%)
Mid-face hypoplasia	-	2/13 (15%)

In the investigated patient, we observed dysmorphic features, developmental delay, speech impairment and sleep disturbances, excessive mouthing behavior, short attention span, hand flapping, fascinating with water, and characteristic EEG and MRI results. The clinical features of our patient are similar to previously published results [4,7,9,12]. The patient's AS symptoms are relatively mild, which correlates well with the previous observations that AS patients with UPD usually have less severe clinical symptoms [8,10,11,13]. The BMI of our patients was > 85%, which correlated well with the previous results of Tan *et al.* (2011) [4] and further confirmed that AS patients with UPD have significantly higher BMI than AS patients with other underlying genetic abnormalities.

The patient was diagnosed with AS at the age of 16 months, earlier than in previous reports of UPD, allowing the parents to be given a correct prognosis and an explanation of delayed neurological developmental as well as the possibility of early interventional therapy. In addition, the parents were counseled that the child is at risk for obesity and its associated complications, which could be managed with lifestyle adjustments. As the aberration was the result of a de novo occurrence, the parents were not counseled on the risk of recurrence for further pregnancies.

## Conclusions

In this paper we report the case of a 16-month-old Hungarian boy affected by AS due to UPD. The early diagnosis of AS has great significance, it allows the parents to be given a correct prognosis and the possibility of early interventional therapy. The detection of UPD and reviewing the previous cases reported in the literature have also pivotal role, since it contributes to the deeper understanding of the phenotype-genotype correlation in AS for non-deletion subclasses. Our data suggest that AS patients with UPD have milder symptoms and higher BMI than AS patients with other underlying genetic abnormalities.

## Methods

Cytogenetic analysis of the child and his parents was carried out with standard methods using G banding with the Cytovision imaging system. The results of the cytogenetic studies suggested UPD, and, therefore, further molecular genetic studies were carried out. Genomic DNA was extracted from venous blood of the index patient (III/1), his parents (II/1, II/2), his grandparents (I/1, I/2, I/3, I/4) and his maternal aunts (II/3, II/4, II/5) [19]. Chromosome 15 segregation analysis with intragenic and extragenic markers for the fibrillin-1 gene was performed for all family members using amplified fragment length polymorphism analysis on an ALFexpress instrument [20]. To determine the molecular background and the recurrence risk, primers for the following microsatellite markers were used in the analysis: D15S119, D15S1028 and MMTS2.

## Consent

Written informed consent was obtained from the patient’s legal guardian for publication of this case report and accompanying images. A copy of the written consent is available for review by the Editor-in-Chief of this journal.

## Abbreviations

AS: Angelman syndrome; PWS: Prader-Willi syndrome; IC: Imprinting center; UBE3A: Ubiquitin protein ligaseE3A gene; UPD: Uniparental disomy; STR: Short tandem repeat; BMI: Body mass index.

## Competing interests

The authors declare that they have no competing interest.
